# A systematic literature review of patient-reported outcome measures used in gout: an evaluation of their content and measurement properties

**DOI:** 10.1186/s12955-019-1125-x

**Published:** 2019-04-11

**Authors:** Carly A. Janssen, Martijn A. H. Oude Voshaar, Peter M. ten Klooster, Tim L. Th. A. Jansen, Harald E. Vonkeman, Mart A. F. J. van de Laar

**Affiliations:** 1 Department of Psychology, Health and Technology, University of Twente, PO BOX 217, 7500 AE Enschede, the Netherlands; 2 Department of Rheumatology, VieCuri Medical Center, Venlo, The Netherlands; 3 Department of Rheumatology and Clinical Immunology, Medisch Spectrum Twente, Enschede, The Netherlands

**Keywords:** Gout, Patient reported outcomes, Measurement properties, systematic literature review

## Abstract

**Background:**

Gout is a common, monosodium urate crystal-driven inflammatory arthritis. Besides its clinical manifestations, patients often also suffer from pain, physical impairment, emotional distress and work productivity loss, as a result of the disease. Patient-reported outcome measures (PROMs) are commonly used to assess these consequences of the disease. However, current instrument endorsements for measuring such outcomes in acute and chronic gout clinical settings are based on limited psychometric evidence. The objective of this systematic literature review was to identify currently available PROMs for gout, and to critically evaluate their content and psychometric properties, in order to evaluate the current status regarding PROMs for use in gout patients.

**Methods:**

Systematic literature searches were performed in the PubMed and EMBASE databases. The methodological quality of included papers was appraised using the COnsensus-based Standards for the selection of health Measurement INstruments (COSMIN) checklist, and evaluation of measurement properties (reliability, responsiveness, construct validity, floor and ceiling effects) was done in accordance with published quality criteria. Item content was appraised by linking health concepts to the International Classification of Functioning Disability and Health (ICF) framework.

**Results:**

In total, 13 PROMs were identified, of which three were targeted specifically at gout patients. The majority of the PROMs were rated positively for content validity. For most instruments, limited evidence was available for construct validity and reliability. Instruments to assess pain scored well on responsiveness and floor and ceiling effects, but not much is known about their reliability in gout.

**Conclusions:**

The physical functioning subscale of the SF-36v2 (Short Form-36 item version 2) is the only PROM that had sufficient supporting evidence for all its psychometric properties. Many of the commonly used PROMs in gout are currently not yet well supported and more studies on their measurement properties are needed among both acute and chronic gout populations.

**Electronic supplementary material:**

The online version of this article (10.1186/s12955-019-1125-x) contains supplementary material, which is available to authorized users.

## Background

Gout is an increasingly prevalent, monosodium urate crystal-driven inflammatory arthritis, commonly presenting as debilitating acute painful flares with associated redness and swelling of the affected joint(s). In some cases a chronic course may develop when increasing crystal deposition is left untreated, leading to visible urate crystal deposits (tophi) and joint damage, as well as extra-articular complications [1]. Along with the clinical manifestations, patients suffering from gout are often confronted with pain, physical impairment, work productivity loss, and emotional distress [2, 3]. Patient-reported outcome measures (PROMs) are commonly used to assess these consequences of gout in a variety of settings [4, 5].

When choosing a specific PROM to use from a number of alternatives, one should take into account the research context, feasibility of the instrument, comparability of scores with relevant earlier work, and the measurement properties of the instrument in the population of interest. Measurement properties are arguably a particularly important factor to consider, since they have a direct bearing on, for example, the ability of a study to demonstrate the desired effects, as well as the required sample size. Therefore, choosing the best instrument from a number of alternatives importantly contributes to the potential for the success of a study. Consequently, endorsements of specific instruments should be based on a comprehensive, critical evaluation of their content and the documented evidence supporting their measurement properties [6].

The OMERACT Gout Special Interest Group has endorsed various patient-reported outcome (PRO) instruments for use in acute and chronic gout clinical research [7–13]. However, these endorsements are based only on the opinions of experts, guided by analyses performed on data from a few selected clinical trials (*n* = 4) and one observational study, as well as a systematic review on the performance of specific measures in previous clinical trials of acute gout [14, 15]. Important measurement properties, such as reliability and validity, are not typically reported on in trial reports, nor can information about these properties necessarily be inferred from the reported results. Also, as information about measurement properties was derived from a small, selected number of studies, new or less popular instruments may have been underappreciated.

To date, no systematic evaluation has been performed of the available evidence supporting the measurement properties of the various PROMs available for use in gout [16, 17]. The objective of this systematic review was to identify all PROMs currently available for gout, and to critically appraise their content and measurement properties, in order to evaluate the current status regarding PROMs validated for use in gout patients, and to identify areas for future research.

## Methods

### Search strategy

To identify all available literature, a systematic literature search was performed in PubMed and EMBASE (database start date, up to August 15, 2017), using a modified, but validated search strategy for papers on measurement properties of PROMs used in gout [18]. The exact search terms are included in the additional material (see Additional file 1). References of included studies and systematic reviews of PROMs found in the search were screened initially by title, and if relevant, abstracts were assessed for potentially relevant papers. Finally, for each included PROM a PubMed search was performed to make sure all papers were included.

### Selection of literature

Inclusion criteria were published articles in which (1) the study population consisted of gout patients and (2) the article reported on the development of a PROM, or the evaluation of one or more of its measurement properties. We excluded (1) conference abstracts and poster presentations, (2) systematic review articles, and (3) articles published in any other language than English.

The titles and abstracts of the retrieved articles were screened independently by two reviewers (MOV and CJ) on relatedness to gout and development or evaluation of a PROM. Any duplicates of articles generated by the search strategy were removed using Microsoft Excel prior to screening. When the title or abstract caused uncertainty pertaining the eligibility criteria, the full-text articles were retrieved and assessed. Disagreements on the eligibility of the article for inclusion were discussed and resolved through consensus. A third reviewer (PtK) was consulted if disagreements remained unresolved. Full-text articles were retained and the final decision on which studies to include were made through consensus after having read the articles (MOV and PtK). Reasons for exclusion were noted and a flow chart of study article selection was prepared according to the Preferred Reporting Items for Systematic Reviews and Meta-Analyses statement [19].

### PROM characteristics

Descriptive characteristics of each instrument were extracted from the included studies or the initial publication of the instrument. The readability of each questionnaire was assessed using the Flesh-Kincaid Grade level-test. A grade level of 6 is recommended by the International Society for Quality of Life Research (ISOQOL) minimum standards for PROMs [20]. Availability of each instrument was determined.

### Assessment of methodological quality

The methodological quality of each included study was assessed using the COnsensus-based Standards for the selection of health Measurement INstruments (COSMIN) checklist [21]. Several deviations from COSMIN checklist were deemed necessary in order to correspond better with advances in psychometric theory or standard practices in gout, and quality of life research [20, 22–26]. An overview of the criteria and our deviations from these are presented in Table 1. Two reviewers (MOV and PtK) independently completed the checklist and final decisions about ratings were arrived at through consensus.

**Table 1:** Quality criteria for rating the measurement properties in accordance with Terwee et al. 2007, and deviations from COSMIN criteria for methodological quality

Measurement property^a^	Definition	Deviations from COSMIN checklist	Threshold for positive rating
Score reliability (single administration)	Classical test theory based estimate of overall proportion of true score variance, calculated from the interitem covariance matrix.	Following Sijtsma et al. 2009, the term “internal consistency” was replaced by single administration reliability. Single administration reliability coefficients were considered to provide information about score reliability.	Reliability coefficient ≥ 0.70.
Score reliability (test-retest)	Classical test theory based estimate of overall proportion of true score variance, obtained from the correlation between repeated measures with same instrument in stable patients.	None, but single administration and test-retest reliability categories were merged in the measurement properties appraisal.	ICC ≥ 0.70.
Construct Validity	The degree to which PRO scores are related to scores of other validated measures in a way that is consistent with theories about how the constructs the measures presume to assess, are related.	None.	At least 75% of the results are in accordance with the hypotheses.
Floor and Ceiling effects	The number of respondents who achieved the lowest or highest possible score.	None.	≤15% of the respondents achieved the highest or lowest possible score.
Responsiveness	The extent to which a PROM can detect changes in the construct being measured over time.	Following the ISOQOL recommendations and Revicky et al. 2008, favorable rating for responsiveness required empirical evidence of changes in scores consistent with a priori expectations of researchers; Either evidenced by score improvement following intervention with known efficacy, or score changes in accordance with expectations derived from external anchors of change (e.g. patient-reported changed overall health status).	Standardized change scores of at least moderate magnitude (e.g., ES / SRM ≥ 0.30 in the expected direction if changes were expected).

^a^For all boxes the reporting standards on missing data were ignored when appraising methodological quality, because such information was rarely reported

*PROMs* patient-reported outcome measures, *COSMIN* consensus-based standards for the selection of health measurement instruments, *ISOQOL* international society for quality of life research, *ICC* intraclass correlation, *PRO* patient-reported outcome, *ES* effect size, *SRM* standardized response mean

### Assessment of measurement properties

The studies that were judged to be of high methodological quality using the COSMIN checklist were used to rate the measurement properties of the included PROM as either good (+) or poor (−), in accordance with quality criteria proposed by Terwee et al. 2007 [26]. Measurement properties of instruments for which only studies of insufficient methodological quality were available were rated as indeterminate (?), or zero (0) when no information was found for that measurement property. In cases where the same PROM was described in various studies of sufficient methodological quality, which resulted in different quality ratings for the same measurement property, the rating was designated as indecisive (+/−). Table 1 gives a description of each rated measurement property, along with the quality criteria applied.

### Content evaluation and assessment of content validity

Health concepts assessed by each PROM were characterized by linking the items to the International Classification of Functioning, disability and Health (ICF) using the 2016 ICF linking rules [27]. As our intention was to compare content between PROMs, we did not link health concepts to the ‘other specified’ or ‘unspecified’ ICF categories. To be rated as having a high content validity (+), ≥ 75% of the health concepts of the PROM had to have been included in either ICF core set [28, 29]. All health items related to emotions were linked to the ICF category ‘b152 emotional functions’.

### Item response theory (IRT)

Although no quality criteria are currently available to judge the quality of studies that used IRT-based analysis, we provided a descriptive review of the results of the included studies that used these methods. As a minimum requirement for methodological quality of the studies, we required that at least 50 patients were included in the study for each item in case of PROMs with dichotomous response categories, or 50 patients for each item step parameter for polytomous data [30]. For articles that used 2-parameter IRT modeling we required a minimum of 250 patients to be included [31]. Furthermore, for a positive rating for methodological quality, the IRT model that was used should be described in sufficiently detail for the reader to understand its parametric structure, or references needed to be included to sources that provide such descriptions. Finally, at least some evidence needed to be presented to support model-data fit.

## Results

**Fig. 1:**
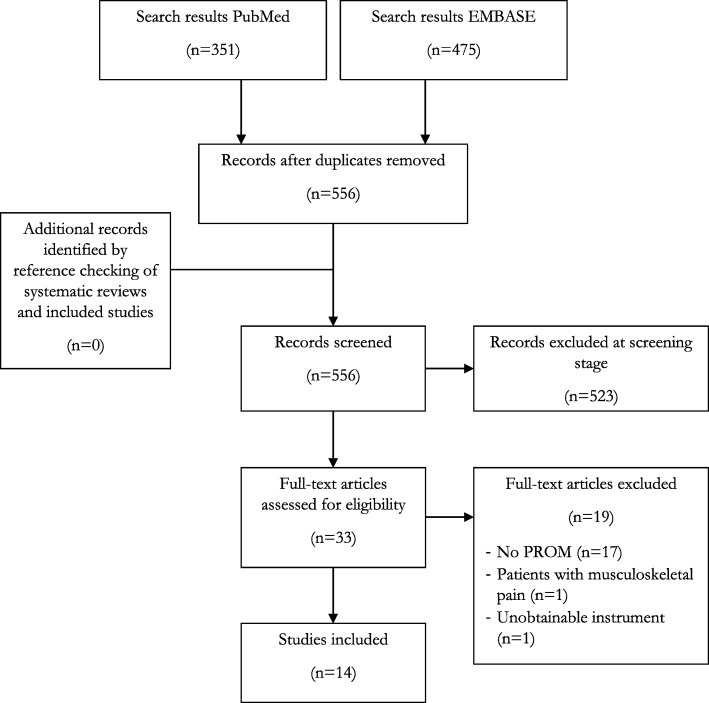
Flow diagram of study selection

The search resulted in 826 hits, of which 556 were screened after removal of the duplicates. After screening of the titles and abstracts, 33 were found to meet the inclusion criteria. Of these, another 19 were excluded, leaving a total of 14 studies for review (see Fig. 1) [15, 32–44]. Reference checking of systematic reviews and of these included papers, or the additional PubMed search of each included instrument, did not result in any additional studies eligible for review.

### Instrument characteristics

The characteristics of the 13 included PROMs are summarized in Table 2 (see Additional file 2 for ICF linking perspectives and categorization of response options). Three of these specifically target gout patients, whereas the remaining instruments target patients with rheumatic diseases or generic populations. Pain and physical function were the most frequently assessed outcome domains. Seven out of eleven (64%) PROMs had Flesch-Kincaid grade level estimates lower than 6, suggesting that their items are easily understood by patients with varying reading proficiency levels (Table 2).

**Table 2:** General characteristics of the included patient-reported outcome measures (PROMs)

			OMERACT core outcome domains		Feasibility	
Instrument	Target population	Subscale (number of items)	Acute gout^a^	Chronic gout^b^	Readability^c^	Availability
*Multidimensional scales*						
SF-36v2 [34–37, 41, 43, 44]	Generic	Physical functioning (10), role-physical (4), bodily pain (2), general health (5), vitality (4), social functioning (2), role-emotional (3), mental health (5)	P, PGA, AL	HRQOL, P, PGA, AL	5.6	License fee may apply
MOS-20 [33]	Generic	Physical function (6), role functional (2), social functioning (1), mental health (5), current perception of health (5), pain (1)	P, PGA, AL	HRQOL, P, PGA, AL	6.5	Freely available
AIMS [33]	Arthritis	Mobility (4), physical activity (5), dexterity (5), household activity (7), social activities (4), activities of daily living (4), pain (4), depression (6), anxiety (6)	P, AL	HRQOL, P, AL	5.6	Freely available
GAQ 2.0 [34, 41, 43]	Gout	GIS (24), consists of 5 subscales: gout concern overall (4), gout medication side effects (2), unmet gout treatment need (3), well-being during attack (11), gout concern during attack (4)	PGA	AGA, HRQOL, P, PGA	7.2^d^	Freely available
*Unidimensional scales*						
HAQ-DI [15, 32, 33, 37–41, 43]	Generic	(43)^e^	AL	AL	4.6	Freely available
HAQ-II [15, 36, 37]	Rheumatic conditions	(10)	AL	AL	4.0	Freely available
TIQ-20 [42]	Tophaceous gout	(20)	–	TB	5.6	Freely available
RA-WIS [36]	RA	(23)	–	–	3.7	License fee may apply
*Single-item PROMs*						
VAS pain^f^ [15, 35]	Multiple	(1)	P	P	3.3^g^	Freely available
Likert pain^h^ [15]	Multiple	(1)	P	P	n/a	Freely available
NRS pain^i^ [15]	Multiple	(1)	P	P	n/a	Freely available
VAS PGA^j^ [15, 35]	Multiple	(1)	PGA	PGA	11.9^k^	Freely available
Physical function NRS^l^ [15]	Gout	(1)	AL	AL	12.7^m^	Freely available

^a^OMERACT mandatory core outcome domains for acute gout are pain (P), joint swelling (JS), joint tenderness (JT), patient global assessment (PGA), activity limitation (AL) [10]

^b^OMERACT mandatory core outcome domains for chronic gout: serum uric acid (sUA), acute gout attack (AGA), tophus burden (TB), Health-related quality of life (HRQOL), activity limitations (AL), pain (P), patient global assessment (PGA) [10]

^c^A Flesch-Kincaid Grade Level score of ≤6 was desired, equivalent to 6th-grade education level or lower in the United States (12 years or lower)

^d^Rated for the GIS section of the GAQ2.0 only

^e^Based upon the HAQ-DI Dutch consensus [51]

^f^100 mm VAS (0 = no to 100 = severe pain)

^g^Rated for the following item “How much pain have you had because of your illness in the past week?” [35]

^h^5-point Likert scale (0 = no pain, 1 = mild pain, 2 = moderate pain, 3 = severe pain, 4 = extreme pain)

^i^11-point NRS (0 = no pain to 10 = extreme pain)

^j^100 mm VAS (0 = very well to 100 = very poor)

^k^Rated for the following item “Considering all the ways that your arthritis affects you, rate how you are doing today on the following scale by placing a vertical mark on the line” [35]

^l^11-point NRS from WPAI:SHP v2.0 (0 = had no effect on my daily activities to 100 = completely prevented me from doing my daily activities) [52]

^m^Rated for the following questionnaire item “During the past 7 days, how much did your gout attack affect your ability to do your regular daily activities, other than work at a job?”

*PROMs* patient-reported outcome measures; *OMERACT* outcome measures in rheumatology, *RA* rheumatoid arthritis, *SF-36v2* Short Form-36 item version 2, *MOS-20* Medical Outcomes Study 20-item Short Form Health Survey, *AIMS* Arthritis Impact Measurement Scales, *GAQ 2.0* Gout Assessment Questionnaire 2.0, *GIS* Gout Impact Scale, *HAQ-DI* Health Assessment Questionnaire-Disability Inde, *HAQ-II* Health Assessment Questionnaire-II, *TIQ-20* 20-item Tophus Impact Questionnaire, *RA-WIS* Rheumatoid Arthritis-Work Instability Scale, *VAS* Visual Analogue Scale, *NRS* Numeric Rating Scale, *PGA* patient global assessment, *−* not applicable, *n/a* not available

### Content description & validity

The results of the ICF-linking exercise revealed that health concepts subsumed under the ICF chapter ‘d4 mobility’ were most frequently addressed in the items of the included PROMs (see Additional file 3). Each included PROM had at least one item related to ‘d4 mobility’. The ‘b1 mental functions’ was the second most popular ICF chapter. This is because all health concepts related to emotional functioning were linked to this chapter. Health concepts related to ‘d8 major life areas’ were also frequently assessed, mainly due to the inclusion of the Rheumatoid Arthritis-Work Instability Scale (RA-WIS). Only three PROMs included content related to environmental factors, particularly the Health Assessment Questionnaire-Disability Index (HAQ-DI), for which scores can be adjusted in case patients need help from others or assistive devices to perform the activities.

Of the in total 32 PROMs, subscales and total scales that were rated, 81% (*n* = 26) met the criteria for a positive rating for content validity (Table 3). However, the role functioning subscales of Medical Outcomes Study 20-item Short Form Health Survey (MOS-20) and Short Form-36 item version 2 (SF-36v2), the Work Productivity and Activity Impairment (WPAI) physical function Numeric Rating Scale (NRS) and several Arthritis Impact Measurement Scales (AIMS) subscales received negative ratings, mainly due to the fact that a large number of their health concepts were too general to be linked to ICF second level categories.

**Table 3:** Quality ratings of the measurement properties of the included instruments

	Truth		Discrimination		
Instrument	Content Validity	Construct validity	Reliability	Responsiveness	Floor and ceiling effects
SF-36v2 [34–37, 41, 43, 44]					
*Physical function*	+	+	+	+	+
*Role physical*	–	?	0	+	–
*Bodily pain*	+	+	0	+	+
*General health*	n/a	?	0	+	+
*Vitality*	+	?	0	+	+
*Social functioning*	+	?	0	+	–
*Role emotional*	+	?	0	–	–
*Mental health*	+	?	0	–	+
*SF-36 PCS*	n/a	?	0	+	+
*SF-36 MCS*	n/a	?	0	–	+
MOS-20 [33]					
*Physical function*	+	?	?	?	0
*Role functioning*	–	?	?	?	0
*Social functioning*	+	?	?	?	0
*Mental health*	+	?	?	?	0
*Health perception*	n/a	?	?	?	0
*Pain*	+	?	?	?	0
AIMS [33]					
*Mobility*	+	?	?	?	0
*Physical activity*	+	?	?	?	0
*Dexterity*	+	?	?	?	0
*Household activities*	–	?	?	?	0
*Social activities*	–	?	?	?	0
*Activities of daily living*	–	?	?	?	0
*Pain*	+	?	?	?	0
*Depression*	+	?	?	?	0
*Anxiety*	+	?	?	?	0
GAQ2.0 [34, 41, 43]					
*Concern overall*	+	?	+/−	+	+
*Medication side effects*	+	?	–	–	+
*Unmet treatment need*	+	?	–	–	+
*Wellbeing during attack*	+	?	+	+	+
*Concern during attack*	+	?	+	–	+
*Total GIS*	n/a	?	+	+	0
HAQ-DI [15, 32, 33, 37–41, 43]	+	+/−	+	+	+/−
HAQ-II [15, 36, 37]	+	+	+	?	–
TIQ-20 [42]	+	+	?	0	+
RA-WIS [36]	+	+	+	0	–
VAS pain [15, 35]	+	+	?	+	+
Likert pain [15]	n/d	?	?	+	+
NRS pain [15]	n/d	?	0	+	+
VAS PGA [15, 35]	n/a	+	0	+	+
WPAI Physical function NRS [15]	–	?	0	+	+

+, good measurement property with sufficient methodological quality; +/−, Indefinite measurement prope rty with sufficient methodological quality; −, poor measurement property with sufficient methodological quality;?, indeterminate quality of measurement properties because of inadequate methodological quality; 0, no information found in the literature; n/a, not applicable; n/d, not definable due to unavailability of questionnaire item;

*AIMS* Arthritis Impact Measurement Scales, *GAQ 2.0* Gout Assessment Questionnaire 2.0, *GIS* Gout Impact Scale, *HAQ-DI* Health Assessment Questionnaire-Disability Index, *HAQ-II* Health Assessment Questionnaire-II, *MOS-20* Medical Outcomes Study 20-item Short Form Health Survey, *SF-36v2* Short Form-36 item version 2, *TIQ-20* 20-item Tophus Impact Questionnaire, *RA-WIS* Rheumatoid Arthritis-Work Instability Scale, *VAS* Visual Analogue Scale, *NRS* Numeric Rating Scale, *PGA* patient global assessment, *WPAI* Work Productivity and Activity Impairment

### Quality rating of measurement properties

Table 3 lists the quality ratings of the psychometric properties of the included PROMs.

#### Construct validity

The methodological quality for construct validity was frequently rated as poor, in the majority of cases because no hypotheses were specified by the authors with respect to expected correlations or mean differences. In studies with explicitly stated hypotheses, positive ratings were generally given, leading to mostly positive ratings for PROMs for which high quality studies of construct validity were available. However, the HAQ-DI was rated as inconclusive as in one study, 78% of hypotheses were confirmed, whereas in another only 61% of hypotheses could be confirmed. For the latter study, hypotheses were not confirmed for some correlations with the subscales of the SF-36v2 (including emotional health, emotional role limitation, social), but also for correlations with outcomes such as the number of gout flares in the past month, Visual Analogue Scale (VAS) for pain, swollen joint count and physician global assessment.

#### Score reliability

The reliability of several multi-item PROMs was supported by high quality studies of single administration reliability. All instruments measuring physical function (HAQ-DI, Health Assessment Questionnaire-II (HAQ-II), SF-36v2 physical functioning subscale) received favorable ratings for reliability, as did the RA-WIS and a couple of the Gout Assessment Questionnaire 2.0 (GAQ2.0) subscales and total scale. The other subscales of the GAQ2.0 were either rated negatively because the reliability coefficient was < 0.70, or as indefinite when studies showed mixed results. The AIMS and MOS-20 were rated as indeterminate because the sample size used for the analysis was inadequate (< 50). None of the studies in which an analysis of test-retest reliability was performed were rated to be of high quality. The 20-item Tophus Impact Questionnaire (TIQ-20) was rated as indeterminate for test-retest despite an intraclass correlation coefficient (ICC) > 0.70 using an otherwise appropriate design, because patients did not appear stable during the two measurement periods. The AIMS and the MOS-20 received an indeterminate rating because an inadequate sample size was used, and the follow-up period of 8 weeks between measurements was deemed too long. For the other six questionnaires, no studies on test-retest reliability were found.

#### Responsiveness

The single-item pain measures (VAS, Likert and NRS) and the bodily pain subscale of the SF-36v2 were demonstrated to be able to detect clinically relevant changes over time. Of the PROMs measuring physical functioning, the HAQ-DI, SF-36v2 (physical functioning subscale, role physical subscale and the physical component summary score) and the single-item WPAI physical function NRS were rated positively, whereas the HAQ-II was rated as indeterminate because it was not clear how patients changed over time. For the same reason, the MOS-20 and AIMS also received an indeterminate rating. The subscales of the SF-36v2 and GAQ2.0 that were rated negatively did so because the demonstrated effect size was considered too small (< 0.30).

#### Floor and ceiling effects

The (sub)scale(s) of the gout-specific GAQ2.0 and the TIQ-20 both showed no floor or ceiling effects. Similarly, the pain instruments were also rated positively, as were the patient global assessment VAS and the general health subscale of the SF-36v2. The instruments for physical functioning showed contradictory results: the HAQ-II had floor or ceiling effects > 15%, the HAQ-DI had indecisive results, and the physical functioning subscale of the SF-36v2 and physical function NRS scale showed no floor or ceiling effects.

### Item response theory (IRT)

There were four articles in which IRT was used. The methodological quality of the first study was rated negatively, because only ~ 24 patients per threshold parameter were included, which makes it unlikely that the estimates of these parameters, which was the subject of their analysis, were stably estimated [39]. In another study, the measurement invariance of the HAQ-DI with respect to diagnosis, was examined [38]. Their results suggest that patients with gout, osteoarthritis and rheumatoid arthritis respond differently to the HAQ-DI categories of walking, dressing, and activities. When these differences in response behavior were controlled for in the model, the authors found that the mean disability scores for the different disease groups were changed slightly. This might impact the validity of cross-diagnostic comparisons using the HAQ-DI. Rasch analysis of the RA-WIS scale provided support for its unidimensionality [36]. Analysis of the locations of the items and persons on the latent measurement continuum revealed that targeting of the scale was supposedly poor, with most of the items clustering together at the middle of the continuum, whereas the distribution of patients was skewed to the right, with a pronounced ceiling effect. Despite this, global reliability was found to be high according to the patient separation index. At last, Rasch analysis was also used in the development of the TIQ-20 [42]. That paper was rated negatively for methodological quality because a longitudinal IRT model was apparently used; however it was not described how the dependencies between the repeated measures were taken into account in the analysis.

## Discussion

### Brief summary

In the current study, we identified and critically reviewed the content and psychometric properties of PROMs currently available for gout, using a systematic approach. This paper can be used for determining areas where further research is required for specific PRO domains and measures in gout, especially regarding their measurement properties.

### Strengths

The comprehensive literature search in various databases, as well as the systematic approach applied during this entire review process, are strengths of this study. In addition, this review is the first to critically review various measurement properties of commonly used PROMs in gout, including the assessment of the methodological quality of studies reporting on these measurement properties. For this purpose, standardized criteria were used to assess both the methodological quality of the included studies using the COSMIN checklist, as well as the quality of the measurement properties using quality criteria that were proposed by ISOQOL and Terwee et al. [20, 21, 26]. Furthermore, the content validity of the included PROMs were comprehensively assessed by linking their items to the ICF using standardized ICF linking procedures [27].

### Weaknesses

There were some limitations to this study. First, our search was developed to find papers that evaluated measurement properties of PROMs used in gout. As a result, we may have missed PROMs used in gout for which no evaluation of the psychometric properties are yet available. For instance, several new generic item banks, for example, those developed for the Patient-Reported Outcomes Measurement Information System project, were not included in this review for that reason [45]. Evaluation of measurement properties of such measures in gout seems very relevant. Moreover, no ICF core set for gout is currently available. The comparative ICF core set we used consisted of the ICF core set of acute inflammatory arthritis and a preliminary ICF core set derived in a recent study in which a core set of gout ICF categories considered relevant by a panel of experts physicians was defined [28, 29]. The results regarding content validity should therefore be considered preliminary and interpreted with some caution. Another limitation to the evaluation of the content of the PROMs is that all health concepts related to emotional functioning (e.g., “Have you been very nervous?”) were linked to a single category, namely ‘b152 emotional functions’. Since health concepts relating to emotional functioning were the second most popular category in the included PROMs, and represented quite diverse emotional experiences, different PROMs could probably be characterized in more detail with respect to the various aspects of emotional functioning they assess. Finally, authors of the included papers were usually insufficiently clear about whether patients had active gouty arthritis, or were studied in the so-called inter-critical periods of the disease. Properties of the included PROMs are likely to differ between these subpopulations, which limits the generalizability of our results. For future studies we recommend that authors provide information on the percentage of patients with active arthritis included in the study.

### Discussion on findings

The results of this study show that various PROMs are available for gout, covering the majority of the outcome domains that have been endorsed by OMERACT for use in clinical studies in this field. Interesting was the absence of studies assessing the properties of PROMs for the OMERACT key outcomes of ‘joint swelling’ and ‘joint tenderness’. Possibly because in many gout clinical studies these outcomes are not applied as a PROM, but are rather assessed by the physician [46, 47]. Nevertheless, patient-reports of these domains have been done in gout clinical studies, so that evaluation of their measurement properties is desired [48, 49]. Also, no studies were found examining the measurement properties of instruments that can be used to derive health utilities for health-economic studies.

Only the physical functioning subscale of the SF-36v2 was rated favorably for all measurement properties in this systematic review. Moreover, in one of the included studies, a direct comparison with the HAQ-DI and HAQ-II showed that it was the only instrument without floor and ceiling effects, suggesting it better targets the disability levels of gout patients [37]. Therefore, current evidence suggests that the SF-36 physical functioning subscale can be recommended for assessing disability in gout. In measuring disability, the HAQ-DI was the only other instrument for which sufficient studies of high quality were available to provide a comprehensive evaluation of its measurement properties. However, this instrument scored inconclusively for construct validity, and floor and ceiling effects. Based on the current evidence, both the VAS and the SF-36v2 bodily pain subscale may be recommended for measuring pain, as almost all measurement properties were supported by high quality studies. However, in general, few studies have yet assessed the psychometric properties of single-item pain measures.

Of the gout-specific PROMs, the health status measuring GAQ2.0 was most extensively evaluated in the literature. Although its subscales showed no floor and ceiling effects, and were all rated as positive for content validity, confirming its items contain health concepts relevant for gout populations, the GAQ2.0 does not cover all recommended OMERACT outcome domains (e.g., no activity limitations scale). This potentially limits its usefulness for gout clinical research purposes. Moreover, the available evidence suggests poor reliability and non-responsiveness to change for half of its subscales, and it was one of the few PROMs with a poorer rating for ease of reading. The overall psychometric appraisal of the GAQ2.0 in this systematic review is in line with previously reported concerns regarding this instrument and therefore we suggest caution in use of this PROM [17]. For assessing health-related quality of life, the current evidence suggests the SF-36v2 may be used as an alternative.

For other instruments, no strong conclusions regarding their psychometric quality were possible, despite the availability of at least one study of most measurement properties for each instrument. With respect to construct validity, this was mostly because authors failed to specify hypotheses about the associations they expected to find. Construct validation is an iterative process in which confidence in the degree to which a PROM actually reflects the construct it intends to measure increases as applications of the measure consistently yield results that would be expected, given theories about how this construct relates to other constructs [50]. Therefore, especially for newly introduced PROMs, proper evaluation of construct validity requires researchers to be specific about expected relations among instruments included in the assessment; taking into account that the relations between the substantive constructs, measurement error and method of measurement all contribute to the observed relations between instruments. For instance, PROMs can be expected to have relatively high intercorrelations, and therefore only limited information about construct validity can be extracted from the finding that significant correlations exist between a number of PROMs. Neither is it the case that higher correlations are always indicative of greater construct validity. Assessments of test-retest reliability in acute gout are complicated by the often rapid improvement that occurs, even without treatment, in the clinical status of patients. This makes it challenging to select a population of stable patients, which led to the many indeterminate ratings in this review. Therefore, for multi-item PROMs, reliability should, in our opinion, be assessed using coefficients that can be calculated from the interitem covariance matrix, such as Cronbach’s alpha.

### Implications for practice

For clinicians working in the field of gout, it may be necessary to understand that little evidence is currently available on the measurement properties of commonly used PROMs, and more importantly, which consequences this may have on outcomes data when poorly supported PROMs are used. In particular as some of the PROMs, for instance the single-item pain PROMs, may be used in daily practice for determining the severity of the pain associated with a gout flare. However, also because evidence from clinical trials, where PROMs are commonly used to collect data, are generally used for developing gout guidelines or management recommendations for in daily clinical practice.

### Implications for research

To ensure high-quality patient-reported outcomes data is collected in gout research it is essential that valid and reliable PROMs are used. Their usage may enhance the feasibility of studies by, for example, creating less measurement error, leading to a smaller required sample size. However, the results from this study show that the measurement properties of the PROMs commonly used in gout clinical research settings are weakly supported. To enhance their position in gout research, we recommend that more evidence on the validity and reliability of PROMs used in gout becomes available. Choosing the most suitable PROM from other alternatives may therefore become easier, and endorsing PROMs for measuring relevant gout outcomes in clinical research, as done by OMERACT, will ideally be based on solid evidence supporting the measurement properties of PROMs.

## Conclusions

In conclusion, the present report presents the results of an evaluation of the content and literature supporting the measurement properties of commonly used PROMs in gout. The results suggest that PROMs are available to assess the majority of the recommended OMERACT core outcome domains for use in clinical research for acute and chronic gout. However, the SF-36 physical functioning subscale is the only PROM that currently meets all the quality criteria we imposed for this review. Many of the commonly used PROMs in this field are currently not yet well supported and more studies on their measurement properties are needed among both acute and chronic gout populations.

## Additional files

Additional file 1: Search strings. Provides the entire search strings applied in both the Pubmed and EMBASE databases for finding appropriate literature. (DOCX 13 kb)

Additional file 2: Linking of instrument subscales to item perspectives and categorization of response options, according to the 2016 ICF linking rules. For each included instrument the response options were categorized, as well as the perspective of the item was determined. This was done so as proposed by the International Classification of Functioning, disability and health linking rules 2016. (DOCX 21 kb)

Additional file 3: Content of the multi- and unidimensional scales used in gout outcome studies according to the International Classification of Functioning (ICF) framework, given as the number, N, and percentage (%) of total health concepts measured. The table provides insight on the content of the patient-reported outcome instruments included in this study, based upon the ICF categories. (DOCX 18 kb)

## Abbreviations

AIMS: Arthritis Impact Measurement Scales
COSMIN: Consensus-based Standards for the selection of health Measurement Instruments
GAQ2.0: Gout Assessment Questionnaire 2.0
HAQ-DI: Health Assessment Questionnaire-Disability Index
HAQ-II: Health Assessment Questionnaire-II
ICC: Intraclass correlation coefficient
ICF: International Classification of Functioning, disability and Health
IRT: Item response theory
ISOQOL: International Society for Quality of Life Research
MOS-20: Medical Outcomes Study 20-item Short Form Health Survey
NRS: Numeric Rating Scale
OMERACT: Outcome Measures in Rheumatology
PRO: Patient-reported outcome
PROM: Patient-reported outcome measure
RA-WIS: Rheumatoid Arthritis-Work Instability Scale
SF-36v2: Short Form-36 item version 2
TIQ-20: 20-item Tophus Impact Questionnaire
VAS: Visual Analogue Scale
WPAI: Work Productivity and Activity Impairment
