# Supplementary material for: A systematic literature review of patient-reported outcome measures used in gout: an evaluation of their content and measurement properties
Source: Health Qual Life Outcomes. 2019 Apr 11;17:63. doi: 10.1186/s12955-019-1125-x (PMC6460780; doi:10.1186/s12955-019-1125-x)
Supplement: Supplementary file 1 — Search strings. Provides the entire search strings applied in both the Pubmed and EMBASE databases for finding appropriate literature. (DOCX 13 kb) [file 12955_2019_1125_MOESM1_ESM.docx]

**Additional file 1**

Initial search string Pubmed:

((gout[MeSH Terms]) OR (gout[Title/Abstract] OR gouty[Title/Abstract])) AND ((valid*[Title/Abstract] OR reliab*[Title/Abstract] OR responsiveness[Title/Abstract] OR discriminative[Title/Abstract] OR Rasch[Title/Abstract] OR "item response theory"[Title/Abstract] OR "sensitivity to change"[Title/Abstract] OR "measurement error"[Title/Abstract]))

Initial search string Embase:

gout:ab,ti OR gouty:ab,ti AND valid*:ab,ti OR reliab*:ab,ti OR responsiveness:ab,ti OR discriminative:ab,ti OR Rasch:ab,ti OR "item response theory":ab,ti OR "sensitivity to change":ab,ti OR "measurement error":ab,ti
