# Supplementary material for: A systematic literature review of patient-reported outcome measures used in gout: an evaluation of their content and measurement properties
Source: Health Qual Life Outcomes. 2019 Apr 11;17:63. doi: 10.1186/s12955-019-1125-x (PMC6460780; doi:10.1186/s12955-019-1125-x)
Supplement: Supplementary file 2 — Linking of instrument subscales to item perspectives and categorization of response options, according to the 2016 ICF linking rules. For each included instrument the response options were categorized, as well as the perspective of the item was determined. This was done so as proposed by the International Classification of Functioning, disability and health linking rules 2016. (DOCX 21 kb) [file 12955_2019_1125_MOESM2_ESM.docx]

**Additional file 2:** Linking of instrument subscales to item perspectives, and categorization of response options, according to the 2016 ICF linking rules

|  |  | **ICF Linking 2016** [1] | |
| --- | --- | --- | --- |
| **Instrument** | **Subscale** | **Item Perspective^a^** | **Response category^b^** |
| SF-36v2 | Physical functioning | DC | IN |
|  | Role-physical | DP | F |
|  | Bodily pain | D | IN |
|  | General health | APP | IN, CoA |
|  | Vitality | DP | F |
|  | Social functioning | DP | IN, F |
|  | Role-emotional | DP | F |
|  | Mental health | DP | F |
| MOS-20 | Physical function | DC | F |
|  | Role functional | DP, DC | F |
|  | Social functioning | DP | F |
|  | Mental health | DP | F |
|  | Current perception of health | APP | IN, CoA |
|  | Pain | D | IN |
| AIMS | Mobility | DP, DC, NoD | CoA |
|  | Physical activity | DC, NoD | CoA |
|  | Dexterity | DC | CoA |
|  | Household activity | DC, NoD | CoA |
|  | Social activities | DP | F |
|  | Activities of daily living | DC, NoD | CoA |
|  | Pain | D | IN, F, DU |
|  | Depression | DP | F |
|  | Anxiety | DP | F |
| GAQ 2.0 | Gout concern overall | DP | IN |
|  | Gout medication side effects | DP | IN |
|  | Unmet gout treatment need | APP | IN |
|  | Well-being during attack | DP, DC | IN, F |
|  | Gout concern during attack | DP | IN |
| HAQ-DI | - | DC, NoD | IN, QA |
| HAQ-II | - | DC | IN |
| TIQ-20 | - | D, DP | CoA |
| RA-WI | - | D, DP | CoA |
| VAS pain | - | D | IN |
| Likert pain | - | D | IN |
| NRS pain | - | D | IN |
| VAS PGA | - | APP | IN |
| Physical function NRS | - | DP, DC | IN |

^a^ Perspectives are descriptive (D), descriptive performance (DP), descriptive capacity (DC), appraisal (APP), need or dependency (NoD)

^b^ Categorization of response options are intensity (IN), frequency (F), duration (DU), confirmation or agreement (CoA), qualitative attributes (QA)

ICF, International Classification of Functioning, disability and Health; SF-36v2, Short Form-36 item version 2; MOS-20, Medical Outcomes Study 20-item Short Form Health Survey; AIMS, Arthritis Impact Measurement Scales; GAQ 2.0, Gout Assessment Questionnaire 2.0; HAQ-DI, Health Assessment Questionnaire-Disability Index; HAQ-II, Health Assessment Questionnaire-II; TIQ-20, 20-item Tophus Impact Questionnaire; RA-WIS, Rheumatoid Arthritis-Work Instability Scale; VAS, Visual Analogue Scale; NRS, Numeric Rating Scale; PGA, patient global assessment; - , not applicable

1. Cieza A, Fayed N, Bickenbach J, Prodinger B. Refinements of the ICF Linking Rules to strengthen their potential for establishing comparability of health information. Disabil. Rehabil. 2016;1–10.
