# Supplementary material for: A systematic literature review of patient-reported outcome measures used in gout: an evaluation of their content and measurement properties
Source: Health Qual Life Outcomes. 2019 Apr 11;17:63. doi: 10.1186/s12955-019-1125-x (PMC6460780; doi:10.1186/s12955-019-1125-x)
Supplement: Supplementary file 3 — Content of the multi- and unidimensional scales used in gout outcome studies according to the International Classification of Functioning (ICF) framework, given as the number, N, and percentage (%) of total health concepts measured. The table provides insight on the content of the patient-reported outcome instruments included in this study, based upon the ICF categories. (DOCX 18 kb) [file 12955_2019_1125_MOESM3_ESM.docx]

**Additional file 3:** Content of the multi- and unidimensional scales used in gout outcome studies according to the International Classification of Functioning (ICF) framework, given as the number, N, and percentage (%) of total health concepts measured.

| ICF category^a^ grouped per component^b^ | HAQ-DI  (N=59) | HAQ-II (N=12) | | SF-36v2 (N=55) | MOS-20 (N=33) | GAQ2.0 (N=34) | TIQ-20 (N=17) | AIMS (N=69) | RA-WIS (N=30) | Total (N=309) |
| --- | --- | --- | --- | --- | --- | --- | --- | --- | --- | --- |
| *Body Functions* | | |  | | | | | | | |
| b1 Mental functions | 0 (0) | 0 (0) | | 14 (25) | 6 (18) | 11 (32) | 4 (24) | 13 (19) | 4 (13) | 52 (17) |
| b2 Sensory functions and pain | 0 (0) | 0 (0) | | 2 (4) | 1 (3) | 2 (6) | 2 (12) | 3 (4) | 2 (7) | 12 (4) |
| b7 Neuromusculoskeletal and movement-related functions | 0 (0) | 0 (0) | | 0 (0) | 0 (0) | 0 (0) | 0 (0) | 1 (1) | 2 (7) | 3 (1) |
| Total Body Functions | 0 (0) | 0 (0) | | 16 (29) | 7 (21) | 13 (38) | 6 (35) | 17 (25) | 8 (27) | 67 (22) |
| *Activities and participation* | | |  | | | | | | | |
| Activities and participation | 2 (3) | 0 (0) | | 8 (15) | 2 (6) | 2 (6) | 0 (0) | 0 (0) | 0 (0) | 14 (5) |
| d1 Learning and applying knowledge | 0 (0) | 0 (0) | | 0 (0) | 0 (0) | 0 (0) | 1 (6) | 1 (1) | 0 (0) | 2 (<1) |
| d2 General tasks and demands | 0 (0) | 0 (0) | | 2 (4) | 0 (0) | 0 (0) | 0 (0) | 0 (0) | 1 (3) | 3 (1) |
| d3 Communication | 0 (0) | 0 (0) | | 0 (0) | 0 (0) | 0 (0) | 0 (0) | 2 (3) | 0 (0) | 2 (<1) |
| d4 Mobility | 18 (31) | 9 (75) | | 14 (25) | 10 (30) | 1 (3) | 2 (12) | 17 (25) | 1 (3) | 72 (23) |
| d5 Self-care | 15 (25) | 1 (8) | | 2 (4) | 4 (12) | 4 (12) | 3 (18) | 6 (9) | 0 (0) | 35 (11) |
| d6 Domestic life | 7 (12) | 2 (17) | | 1 (2) | 2 (6) | 2 (6) | 2 (12) | 4 (6) | 0 (0) | 20 (6) |
| d7 Interpersonal interactions and relationships | 0 (0) | 0 (0) | | 0 (0) | 0 (0) | 0 (0) | 1 (6) | 3 (4) | 0 (0) | 4 (1) |
| d8 Major life areas | 0 (0) | 0 (0) | | 8 (15) | 4 (12) | 3 (9) | 1 (6) | 1 (1) | 20 (67) | 37 (12) |
| d9 Community, social and civic life | 0 (0) | 0 (0) | | 4 (7) | 4 (12) | 5 (15) | 1 (6) | 4 (6) | 0 (0) | 18 (6) |
| Total Activities and Participation | 42 (71) | 12 (100) | | 39 (71) | 26 (79) | 17 (50) | 11 (65) | 38 (55) | 22 (73) | 207 (67) |
| *Environmental factors* | | |  | | | | | | | |
| Environmental factors | 2 (3) | 0 (0) | | 0 (0) | 0 (0) | 0 (0) | 0 (0) | 0 (0) | 0 (0) | 2 (<1) |
| e1 Products and technology | 13 (22) | 0 (0) | | 0 (0) | 0 (0) | 4 (12) | 0 (0) | 1 (1) | 0 (0) | 18 (6) |
| e3 Support and relationships | 2 (3) | 0 (0) | | 0 (0) | 0 (0) | 0 (0) | 0 (0) | 13 (19) | 0 (0) | 15 (5) |
| Total Environmental factors | 17 (29) | 0 (0) | | 0 (0) | 0 (0) | 4 (12) | 0 (0) | 14 (20) | 0 (0) | 35 (11) |

^a^ ICF first level categories

^b^ ICF components include Body Functions, Activities and Participation, and Environmental Factors. The component Body Structures was not represented.

HAQ-DI, Health Assessment Questionnaires-Disability Index; HAQ-II, Health Assessment Questionnaires-II; SF-36v2, Short Form-36 item version 2; MOS-20, Medical Outcomes Study 20-item Short Form Health Survey; GAQ, Gout Assessment Questionnaire; TIQ-20, 20-item Tophus Impact Questionnaire; AIMS, Arthritis Impact Measurement Scales; RA-WIS, Rheumatoid Arthritis-Work Instability Scale
